# Sleep fragmentation, impaired glymphatic clearance, and long-term cognitive impairment after critical illness

**DOI:** 10.62675/2965-2774.20260042

**Published:** 2026-03-16

**Authors:** Fawaz Alshammari, Samantha A. Keil, Mary Elizabeth Wilcox

**Affiliations:** 1 University of Alberta Faculty of Medicine and Dentistry Department of Critical Care Medicine Alberta Canada Department of Critical Care Medicine, Faculty of Medicine and Dentistry, University of Alberta, Alberta, Canada.; 2 Brain Health Imaging Institute Weill Cornell Medicine Department of Radiology New York NY USA Department of Radiology, Brain Health Imaging Institute, Weill Cornell Medicine, New York, NY, USA.; 3 University of Alberta Neurosciences and Mental Health Institute Alberta Canada Neurosciences and Mental Health Institute, University of Alberta, Alberta, Canada.

**Keywords:** Sleep, Sleep, slow-wave, Intensive care units, Glymphatic system

## Abstract

Sleep disruption is nearly universal during critical illness and is increasingly linked to long-term cognitive impairment among intensive care unit survivors. Advances in neuroscience have highlighted the glymphatic system – a brain-wide perivascular network that clears metabolic waste such as β-amyloid and phosphorylated tau – as a plausible mechanism connecting sleep disturbance to adverse neurocognitive outcomes. Glymphatic transport is highly state-dependent, functioning optimally during slow-wave sleep and diminishing with wakefulness or fragmented sleep. This invited review synthesizes preclinical and human evidence showing that sleep fragmentation and loss of slow-wave sleep impair cerebrospinal fluid–interstitial fluid exchange, promote accumulation and spread of tau and other neurotoxic proteins, and may accelerate neurodegenerative trajectories. We discuss how commonly used sedatives may induce unconsciousness without reproducing the coordinated neuromodulatory and neurovascular conditions of natural slow-wave sleep, creating "pseudo-sleep" that may fail to support metabolic clearance. We propose glymphatic dysfunction as an integrative pathway linking intensive care unit sleep disruption, inflammation, and sedative exposure to persistent deficits in memory, attention, and executive function after critical illness.

## INTRODUCTION

Sleep has long been recognized as essential for cognitive and physiological restoration. However, recent discoveries about the brain's glymphatic system have transformed our understanding of sleep's role in the clearance of neurotoxic waste. The glymphatic pathway functions as a coordinated network of perivascular channels that facilitates cerebrospinal fluid (CSF) influx and interstitial fluid (ISF) efflux, thought to facilitate the clearance of metabolic by-products, including β-amyloid and phosphorylated tau.^(1)^ Importantly, glymphatic activity is state-dependent: it operates optimally during slow-wave sleep (SWS) and is markedly reduced during wakefulness or disrupted sleep.^(2)^ In critically ill patients, severe sleep disruption, altered neuromodulation, and prolonged sedation may impair glymphatic clearance, potentially contributing to persistent cognitive impairment after critical illness.

## THE GLYMPHATIC SYSTEM AND ITS DEPENDENCE ON SLOW-WAVE SLEEP

The glymphatic system relies on convective exchange driven by arterial pulsatility, astroglial aquaporin-4 (AQP4) channels, and neuromodulatory shifts associated with deep sleep. During SWS, reduced cortical norepinephrine release leads to a marked expansion of the interstitial space, up to 60%, which facilitates CSF flow through para-arterial pathways and enhances clearance of neurotoxic proteins.^(2)^ Furthermore, electrophysiological slow waves synchronize neuronal down-states with vascular pulsations, generating large-scale hemodynamic oscillations that directly correlate with CSF influx in human studies.^(3)^ These findings illustrate that SWS is not merely a restorative period but an active, physiological state in which the brain undergoes metabolic cleansing.

When SWS is disrupted, whether by sleep fragmentation, pain, mechanical ventilation, or sedatives, the glymphatic system may be impaired. This provides a possible mechanistic explanation for growing evidence linking impaired or hindered SWS to the accumulation of tau and amyloid-β, early neurodegeneration, and cognitive decline.^(4)^

## SLEEP FRAGMENTATION AS A DISRUPTOR OF GLYMPHATIC FUNCTION

Sleep fragmentation, characterized by frequent arousals, disrupts normal sleep architecture and can have significant neurophysiological effects independent of total sleep duration. Brief awakenings increase norepinephrine levels, constricting the interstitial space and inhibiting glymphatic flow.^(2)^ Preclinical models show that fragmented sleep accelerates extracellular tau release, promotes tau spread across synaptically connected brain regions, and leads to impairments in memory and spatial navigation.^(5)^ Human studies corroborate these findings: sleep fragmentation, particularly from disorders such as obstructive sleep apnea, is associated with elevated CSF tau levels and increased risk of cognitive decline and dementia.^(6)^

In the intensive care unit (ICU), environmental factors such as noise, continuous lighting, frequent clinical interventions, pain, respiratory distress, and *delirium* disrupt normal sleep, resulting in an irregular pattern dominated by stages 1 and 2, with little or no SWS or rapid eye movement (REM) sleep.^(7)^ Critically ill patients may also lose normal ultradian sleep organization altogether. Polysomnography often demonstrates profound suppression of both SWS and REM sleep.^(8)^ This continuous fragmentation may impair glymphatic clearance during a period when injured or inflamed brains most need efficient waste removal.

Chronic suppression of glymphatic clearance may accelerate the accumulation of phosphorylated tau, which propagates trans-synaptically and compromises memory circuits. Animal models show that impaired sleep or reduced SWS increases tau burden in the hippocampus and entorhinal cortex, key regions vulnerable in both Alzheimer's disease and post-ICU cognitive impairment.^(5)^ Human studies demonstrate that decreased SWS correlates with increased tau PET binding and predicts future cognitive decline.^(9)^

## SEDATIVES, ANESTHESIA, AND PSEUDO-SLEEP: IMPLICATIONS FOR GLYMPHATIC SUPPRESSION

Sedatives are widely used in critical care for comfort, ventilator synchrony, and lung protective ventilation strategies (Table 1). However, many commonly used sedatives, including benzodiazepines, propofol, and opioids induce states of unconsciousness while supressing the neuromodulatory and neurovascular conditions characteristic of natural slow-wave sleep.^(8)^ Crucially, these agents do not produce the sustained suppression of noradrenergic tone, coordinated cortical states, and stable coupling between neuronal, vascular, and respiratory rhythms thought to facilitate glymphatic CSF-ISF exchange. Further, while some anesthetics, such as dexmedetomidine,^(10)^ generate electroencephalography (EEG) patterns resembling SWS, it is unclear if they fully recapitulate the environment required for optimal glymphatic activation.^(8)^

**Table 1:** Proposed contributors to glymphatic dysfunction and cognitive impairment after critical illness

ICU-related factor	Sleep/neurophysiology effect	Proposed glymphatic mechanism	Downstream consequence
ICU environment (noise, light, interventions, pain)	Sleep fragmentation; reduced SWS/REM	↑ arousals → ↑ noradrenergic tone → reduced interstitial space expansion → ↓ CSF influx/ISF efflux	Reduced clearance of neurotoxic metabolites
Sleep fragmentation	Loss of consolidated SWS	Disrupts SWS-dependent glymphatic flow	Associated with ↑ tau release/spread; memory impairment
Loss of slow-wave sleep	Reduced slow oscillations; impaired restorative sleep	Reduced interstitial space expansion (∼60% during SWS) and ↓ CSF oscillations	↑ amyloid-β and p-tau accumulation risk
Sedation ("pseudo-sleep")	EEG changes may not replicate natural sleep neuromodulation	May not reproduce synchronized cortical down-states / noradrenergic shifts needed for glymphatic activation	Persistent impaired waste clearance despite apparent sleep
Opioid exposure / ventilatory effects	Depressed respiratory drive, CO_2_ retention	May ↓ respiratory pulsatility contribution to perivascular CSF movement; possible ↑ ICP	Impaired clearance efficiency.
Critical illness inflammation / BBB dysfunction	Astrocyte dysfunction, microglial activation	AQP4 mislocalization and impaired astroglial endfeet polarity → ↓ convective transport	Reduced clearance during increased metabolic burden
Mechanical ventilation / hemodynamic instability	Altered pulsatility and coupling	May ↓ arterial pulsatility driving glymphatic convection	↓ clearance capacity
Post-ICU sleep disturbance	Persistent sleep abnormalities after discharge	May contribute to suppression of glymphatic clearance	Sustained tau accumulation trajectory

ICU - intensive care unit; SWS - slow wave sleep; REM - rapid eye movement; CSF - cerebrospinal fluid; ISF - interstitial fluid; EEG - electroencephalography; CO_2_ - carbon dioxide; ICP - intracranial pressure; BBB - blood brain barrier; AQP4 - aquaporin 4; p-tau - phosphorylated tau protein.

At different phases of critical illness, mechanically ventilated patients may require continuous and possibly prolonged periods of intravenous sedation.^(11)^ The resulting state of "pseudo-sleep" may give the appearance of rest while depriving the brain of the physiological conditions needed for effective waste clearance. This may resultantly set the stage for the accumulation and inefficient clearance of neurotoxic bioproducts, potentially slowing clinical improvement or accelerating the neurodegenerative process after critical illness.^(12)^

## CRITICAL ILLNESS, NEUROINFLAMMATION, AND THEIR INTERACTION WITH GLYMPHATIC DYSFUNCTION

Critical illness is itself a pro-inflammatory state. Systemic inflammation disrupts blood-brain barrier integrity, alters astrocytic endfeet polarity, and reduces AQP4 localization, thereby compromising glymphatic transport.^(13)^ At the same time, sepsis, acute respiratory distress syndrome (ARDS), and multi-organ failure lead to oxidative stress, microglial activation, and increased neuronal metabolic burden.^(14,15)^ The brain, therefore, generates more metabolic waste precisely when its clearance pathways may be the most impaired.

**Figure 1:**
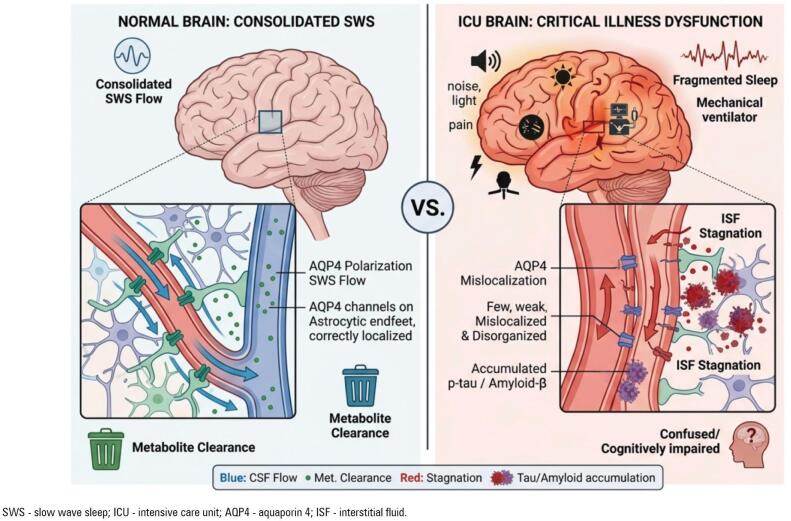
Proposed comparative mechanism of glymphatic function in normal and critical illness states.

*Delirium*, prevalent in 50 - 80% of ICU patients, is associated with inflammatory cytokines and disrupted sleep architecture,^(16,17)^ reinforcing a cycle of neurophysiological dysfunction. The convergence of these insults – neuroinflammation, sleep loss, sedative exposure, and mechanical ventilation – produces an environment in which glymphatic impairment may become severe and sustained (Figure 1).

*Generated by Nano Banana 2 AI under Google AI studio.

Long-term cognitive impairment is one of the most common sequelae of critical illness, affecting 30 - 50% of ICU survivors months to years after discharge.^(18-21)^ Many exhibit deficits in attention, executive function, and memory resembling early Alzheimer-type dementia. While hypoxia, inflammation, and microvascular injury contribute, glymphatic dysfunction provides an integrative mechanism linking sleep disruption to neurodegeneration.

Finally, ICU survivors exhibit persistent sleep abnormalities months after discharge,^(22)^ raising the possibility that impaired glymphatic function continues beyond the acute phase. If so, this may create a long-term trajectory of cumulative tau accumulation, synaptic dysfunction, and progressive cognitive impairment.

## CONCLUSION

Sleep fragmentation and loss of slow-wave sleep profoundly disrupt glymphatic clearance, increasing vulnerability to neurotoxic protein accumulation. In critically ill patients, environmental disturbances, neuroinflammation, sedative exposure, and mechanical ventilation create a perfect storm that may suppress glymphatic function at a time when efficient waste clearance is crucial. Mounting evidence suggests that future studies investigating the high rates of long-term cognitive impairment and incident dementia observed after critical illness might be driven, in part, by impaired glymphatic activity. Understanding these pathways underscores the need for intensive care unit practices that preserve natural sleep physiology, minimize sedative exposure when possible, and support neuroprotective recovery.

## Data Availability

The data cannot be made publicly available as it is a summary review.
